# The mechanism of high-quality logistics in rural industrial upgrading—A dual moderation perspective of technological innovation and digital finance

**DOI:** 10.1371/journal.pone.0344923

**Published:** 2026-05-04

**Authors:** Borui Yan, Bo Yao, Qianyu Guo, Xiaonan Zhang

**Affiliations:** 1 School of Economics and Management, Xianyang Normal University, Xianyang, Shaanxi, China; 2 Department Computer Science and Engineering, Langfang Polytechnic Institute, Langfang, Hebei, China; Universiti Malaysia Sabah, MALAYSIA

## Abstract

High-quality logistics development plays a crucial role in upgrading the rural industrial structure. It aims to explore the impact mechanism and underlying logic of high-quality logistics development on the upgrading of rural industry. By constructing an empirical model and utilizing statistical data from 30 provinces in China from 2011 to 2022, it analyzes the role of high-quality logistics development in promoting rural industrial upgrading. Furthermore, it examines the mediating, moderating, and threshold effects of factors such as regional industrial structure, fiscal support for technological innovation, digital finance, agricultural labor productivity, and the urban-rural income gap. The results indicate that high-quality logistics development significantly promotes rural industrial upgrading, and this effect remains robust after incorporating control variables. Moreover, regional industrial structure acts as a mediating effect to strengthen this positive impact; enhanced fiscal support for technological innovation and digital finance further amplify the effect; and agricultural labor productivity and the urban-rural income gap present significant threshold effects, leading to heterogeneous impacts of high-quality logistics development across different levels of these factors.

## 1 Introduction

In the process of deep integration between the digital economy and modern agriculture, the logistics industry is undergoing a paradigm shift from traditional factor-driven growth to a dual-driven model powered by technology and capital. Particularly under the strategic framework of establishing a “dual circulation” development pattern, the synergistic mechanism between high-quality logistics development and the upgrading of the regional industrial structure has become a key issue for overcoming barriers to urban-rural factor mobility and fostering endogenous rural growth [1]. A review of relevant domestic and international literature reveals a close relationship between the logistics industry and industrial development. The logistics industry can reduce transaction costs, improve market efficiency, and provide strong support for industrial development [2]. With the advancement of technology and the upgrading of logistics services, it plays an increasingly prominent role in industrial upgrading and structure optimization. In rural areas specifically, it is essential for overcoming the constraints of traditional agricultural production and stimulating the growth of agricultural processing and related services [3]. However, existing studies have confirmed the positive impact of logistics infrastructure on rural production [4], two critical theoretical gaps remain. First, there is a lack of mechanistic explanation for the synergy between technological innovation and digital finance within the logistics-agriculture system, making it difficult to account for differentiated upgrading effects of identical logistics investments across regions. Second, the threshold effects of technological penetration and capital allocation have been overlooked, leading to inefficiencies in the policy transmission of “logistics empowering agriculture”. To address these gaps, this study constructs an analytical framework incorporating dual moderating effects to answer two core questions: (1) How do technological innovation and digital finance reshape the impact pathway of logistics on rural industrial upgrading through complementary mechanisms? (2) Under different technology-capital endowment conditions, what nonlinear characteristics does the marginal contribution of logistics quality exhibit?

## 2 Literature review and hypotheses

### 2.1 The impact of high-quality development of the logistics industry on rural industrial upgrading

In terms of the connotation of high-quality development, it is not quantitative growth, but the simultaneous improvement of economic quality and efficiency. Rooted in global sustainable and inclusive growth pursuits, this concept aligns with the UN Sustainable Development Goals (SDGs)—notably SDG 12 (Responsible Consumption and Production) and SDG 8 (Decent Work and Economic Growth)— emphasizes green development and long-term resilience, ensuring its global relevance beyond regional contexts. Thus, high-quality logistics development entails balancing efficiency and quality with sustainable principles, encompassing not only efficiency but also service reliability, green sustainability, and supply chain adaptability. It is reflected in improved efficiency, cost reduction, service optimization and comprehensive enhancements in technological innovation, green sustainability, and supply chain coordination [5]. Traditional rural industries, dominated by low value-added, short chain agriculture with weak resilience, undergo upgrading through technological innovation, restructuring, and brand development—transforming into high-value-added, high-tech, competitive sectors. This upgrading manifests in the integration of agriculture with processing, tourism, e-commerce(e.g., agricultural product deep-processing clusters, cultural tourism chains), enhanced technological content, (smart agriculture, digital rural industries), and improved market competitiveness, ultimately achieving a qualitative leap in rural economic development and optimizing the rural industrial structure.

As a crucial component of the modern service industry, the logistics industry is closely linked to rural industrial development. High-quality logistics services play a pivotal role in accelerating the circulation of agricultural products, expanding sales channels, and enhancing their market competitiveness. Specifically, high-quality logistics development can directly facilitate rural industrial upgrading in the following ways: (1) Enhancing the efficiency of agricultural product circulation: An efficient logistics system reduces the time from production to consumption, minimizes losses, and improves the freshness and quality of agricultural products. This strengthens their market competitiveness and promotes the branding and standardization of agricultural goods. (2) Promoting rural industrial upgrading: The development of the logistics industry stimulates the growth of related sectors, including warehousing, processing, and packaging, thereby extending the agricultural product value chain. It also fosters the integration of primary, secondary, and tertiary industries in rural areas, ultimately contributing a more comprehensive rural industrial system. (3) Optimizing resource allocation: High-quality logistics services help overcome geographical constraints, enabling the more effective allocation of resources at a broader scale. This not only facilitates the efficient utilization of internal rural resources but also attracts external capital, technology, and talent, providing strong momentum for rural industrial upgrading. (4) Strengthening risk resistance: A well-developed logistics network and information system enhance rural industries’ responsiveness to market fluctuations. By mitigating risks associated with information asymmetry and logistics inefficiencies, they help ensure the stable development of rural industries. Based on the above analysis, this study proposes Hypothesis 1:

H1: High-quality logistics development has a significant impact on rural industrial upgrading.

### 2.2 The mediating role of industrial structure

The level of regional industrial structure is a comprehensive indicator that reflects the proportional relationships, interconnections, and upgrading trends among various industries (primary, secondary, tertiary industries, and their subdivisions) within a specific region. By reviewing relevant literature, we find that the development of the logistics industry has a significant optimization effect on the regional industrial structure. On the one hand, as a bridge connecting production and consumption, the efficient operation of the logistics industry reduces transaction costs and accelerates market responsiveness, thus promoting the collaborative development of various industries within the region [6]. On the other hand, the development of the logistics industry is often accompanied by technological and business model innovations, which can permeate other industries and drive the upgrading and transformation of the industrial structure [7]. Additionally, some studies suggest that the development of the logistics industry can extend and expand related industrial chains, thereby fostering the diversification and upgrading of the regional economy [8]. As a core component of the modern service industry, high-quality logistics development can profoundly influence and optimize the regional industrial structure. Specifically, high-quality logistics development enhances the overall economic efficiency of the region, promotes the optimal allocation and efficient utilization of regional resources, and provides more market opportunities and development space for rural industries, guiding them toward higher value-added and more technology-intensive directions. Based on this, this study proposes Hypothesis 2:

H2: High-quality logistics development can promote rural industrial upgrading by improving the level of regional industrial structure.

### 2.3 The moderating effects of regional fiscal support for technological innovation and digital finance

#### 2.3.1 The moderating effect of regional fiscal support for technological innovation.

A review of relevant literature reveals that technological innovation is a critical driver of industrial upgrading and transformation. Technological advancements not only increase the technological content and added value of industries but also promote improvements across related industrial chains through technology diffusion and spillover effects [9]. Meanwhile, as a bridge connecting production and consumption, high-quality logistics development is also highly dependent on technological innovation. Technological progress can enhance logistics efficiency, reduce costs, and improve the intelligence and customization of logistics services [10]. Regional fiscal support for technological innovation refers to the resources and enabling environment provided by local governments or relevant entities to facilitate technological innovation in logistics and rural industries. This support may take the form of fiscal subsidies (such as special funds for logistics technology research and development), policy guidance (such as construction plans for smart logistics parks), and platform construction (such as rural logistics technology collaborative innovation centers). Furthermore, studies indicate that the level of regional fiscal support for technological innovation directly influences the efficiency of technology commercialization and application, which in turn affects overall economic development and the upgrading of the industrial structure [11].

Regional fiscal support for technological innovation plays a pivotal role in driving technological progress and innovation. In the context of high-quality logistics industry development, local governments can stimulate logistics technology innovation and upgrading by increasing investments in research and development. Technologies such as intelligent warehousing, automated sorting, and IoT-based tracking can significantly enhance logistics efficiency and service quality [11]. Enhanced fiscal support for technological innovation fosters continuous advancement in logistics technologies, enabling the logistics industry to serve rural industries more efficiently and intelligently. This, in turn, reduces logistics costs for rural industries and improves logistics performance, creating favorable conditions for rural industrial upgrading. Based on this, it proposes Hypothesis 3:

H3: Regional fiscal support for technological innovation positively moderates the impact of high-quality logistics industry development on rural industrial upgrading.

#### 2.3.2 The moderating role of digital finance.

The level of digital finance refers to the coverage, depth of application, and innovation degree of digital financial services within a region, including the popularization of mobile payments, innovations in supply chain finance, and big data credit services. At its core, digital finance leverages digital technologies to overcome the temporal, spatial, and risk management limitations of traditional financial services in rural areas. In recent years, research on the impact of digital finance on economic development has grown substantially. Digital finance facilitates the efficient allocation of capital and accelerates economic growth by reducing financial service barriers, improving service efficiency, and expanding financial coverage [12]. In the logistics industry, the application of digital finance has driven innovations in logistics-related financial services, such as supply chain finance and blockchain-based solutions, providing the logistics sector with more convenient and efficient financing and settlement services [13]. At the same time, digital finance offers rural industries additional financing channels and access to market information, helping them overcome capital constraints and information asymmetry, thereby accelerating their development [14]. During the process of high-quality logistics industry development, the widespread adoption of digital finance provides rural industries more accessible and cost-effective financing channels and payment solutions, reducing their financing costs and operational risks. First, digital finance enhances the accessibility and convenience of financial services, lowering the cost and difficulty of obtaining financial support for both rural industries and the logistics sector, thereby promoting their coordinated development. Second, the broad application of digital finance facilitates information flow and sharing, enabling the logistics industry to better understand market demand and trends. This allows logistics services to operate more precisely and efficiently, assisting rural industries in adjusting their structure and upgrading in response to market needs. Based on this, it proposes Hypothesis 4:

H4: The level of digital finance positively moderates the impact of high-quality logistics industry development on rural industrial upgrading.

### 2.4 The threshold effects of agricultural labor productivity and the urban-rural income ratio

#### 2.4.1 The threshold effect of agricultural labor productivity.

Agricultural labor productivity refers to the output value created per unit of labor input in the agricultural production process. It reflects the efficiency and modernization level of agricultural production and is a core indicator for measuring the quality of agricultural development. Existing literature generally recognizes improvements in agricultural labor productivity as a key driver of agricultural modernization and rural industrial upgrading [15]. At the same time, the development of the logistics industry plays a critical role in enhancing agricultural production efficiency. By optimizing logistics network, reducing costs, and improving operational efficiency, the logistics sector can support the advancement of agricultural production to higher level [16]. The existence of a threshold effect of agricultural labor productivity is underpinned by two core mechanisms: the “resource matching effect” and the “technology absorption threshold”. On the one hand, high-quality logistics development (e.g., intelligent scheduling systems, cold chain logistics networks) requires a corresponding level of agricultural production capacity to match its service efficiency—this constitutes the “resource matching effect”. When agricultural labor productivity is below a certain threshold, production is often constrained by low-efficiency modes (e.g., small-scale manual farming, limited mechanization), resulting in restricted per capita output. At this stage, even if high-quality logistics services are available, producers lack the necessary resources (e.g., funds for standardized packaging, sufficient scale for batch transportation) to fully utilize them. For example, smallholder farmers with low labor productivity may be unable to afford cold chain logistics for fresh products, resulting in logistics services being “available but not usable”, thus limiting the role of logistics in promoting rural industrial upgrading. On the other hand, the spillover effects of high-quality logistics (e.g., market information transmission, technical guidance in circulation) depend on a certain level of agricultural technology to be effectively absorbed—this is the “technology absorption threshold”. When agricultural labor productivity exceeds this threshold, it generally signals the adoption of advanced agricultural technologies (e.g., precision farming, automated irrigation) and improved farmer literacy. At this point, producers can leverage high-quality logistics to access larger markets (e.g., using logistics tracking systems to adjust planting plans based on market feedback) and transition from low-value-added primary product sales to high-value-added processing or branded operations, thereby maximizing the impact of logistics on industrial upgrading.

When agricultural labor productivity reaches a sufficient level, the agricultural sector can more effectively absorb and transform the spillover effects of logistics development. Specifically, high productivity implies higher technical standards and stronger market competitiveness, enabling producers to adopt advanced logistics technologies and service models to enhance production efficiency and product quality. Consequently, in the context of the high-quality development, rural areas with high agricultural labor productivity are more likely to achieve industrial optimization and upgrading. Conversely, if productivity is low, producers may not fully capitalize on logistics industry advancements due to limitations in technology and capital. In such cases, although logistics development provides external support to rural industries, its effect on industrial upgrading may be limited. Based on this, it proposes Hypothesis 5:

H5: There exists a threshold effect of agricultural labor productivity in the impact of high-quality logistics development on rural industrial upgrading.

#### 2.4.2 The threshold effect of the urban-rural income gap.

The urban-rural income gap refers to the absolute difference or relative ratio between the per capita disposable income of rural and urban residents. It reflects the balance of economic development between urban and rural areas, and embodies the consumption capacity of rural residents as well as the potential driving force for factor flows between urban and rural regions. It is an important determinant of rural economic development. Against the backdrop of high-quality logistics development, changes in the urban-rural income gap may also exert a threshold effect on rural industrial upgrading. First, from a market demand perspective, high-quality logistics (e.g., cold chain and integrated supply chain services) requires sufficient scale demand to be sustainable. When the income gap exceeds a certain threshold, rural consumption is limited to basic needs, lacking demand for high-value-added agricultural products or diversified logistics services. This reduces the profitability and coverage of high-quality logistics, thereby limiting its ability to drive rural industrial upgrading. Conversely, when the gap is below the threshold, rural consumption capacity increases, generating “demand-pull” effects that promote the circulation of high-value-added products and facilitate rural industrial upgrading. Second, from a factor supply perspective, a large income gap induces outflows of talent and capital, leaving insufficient factors (e.g., e-commerce professionals, logistics infrastructure funds) to support industrial upgrading. Even with high-quality logistics facilities, their effectiveness is constrained without adequate supporting factors. A narrower income gap attracts factor inflows to rural areas, which, when combined with high-quality logistics, forms synergies that drive industrial upgrading.

Existing literature indicates that the urban-rural income gap is a key factor influencing the coordinated development of regional economies [17]. On one hand, narrowing the gap promotes rural economic development and social progress, enhances the consumption capability and investment willingness of rural residents, and thus provides a broader market space for the expansion of the logistics industry in rural areas [18]. On the other hand, high-quality logistics development can reduce logistics costs and improve operational efficiency, thereby enhancing the market circulation and value-added of agricultural products, and potentially alleviating the urban-rural income gap to some extent [19]. However, few studies have examined how the urban-rural income gap, as a threshold factor, influences the role of logistics industry development in rural industrial upgrading. Specifically, when the income gap is small, it indicates relatively favorable economic development and social welfare in rural areas, with stronger consumption capacity and investment willingness among rural residents. In this context, high-quality logistics development can more effectively penetrate rural areas, providing convenient and efficient logistics services that promote rural industrial upgrading. In contrast, when the income gap is large, rural areas may face resource scarcity, inadequate infrastructure, and brain drain, which constrain the expansion and deepening of the logistics industry. Even if the logistics industry achieves high-quality development overall, its impact on rural industrial upgrading may be significantly weakened due to the restrictive effect of the urban-rural income gap. Based on this, it proposes Hypothesis 6:

H6: There exists a threshold effect of the urban-rural income gap in the impact of high-quality logistics industry development on rural industrial upgrading.

**Fig 1:**
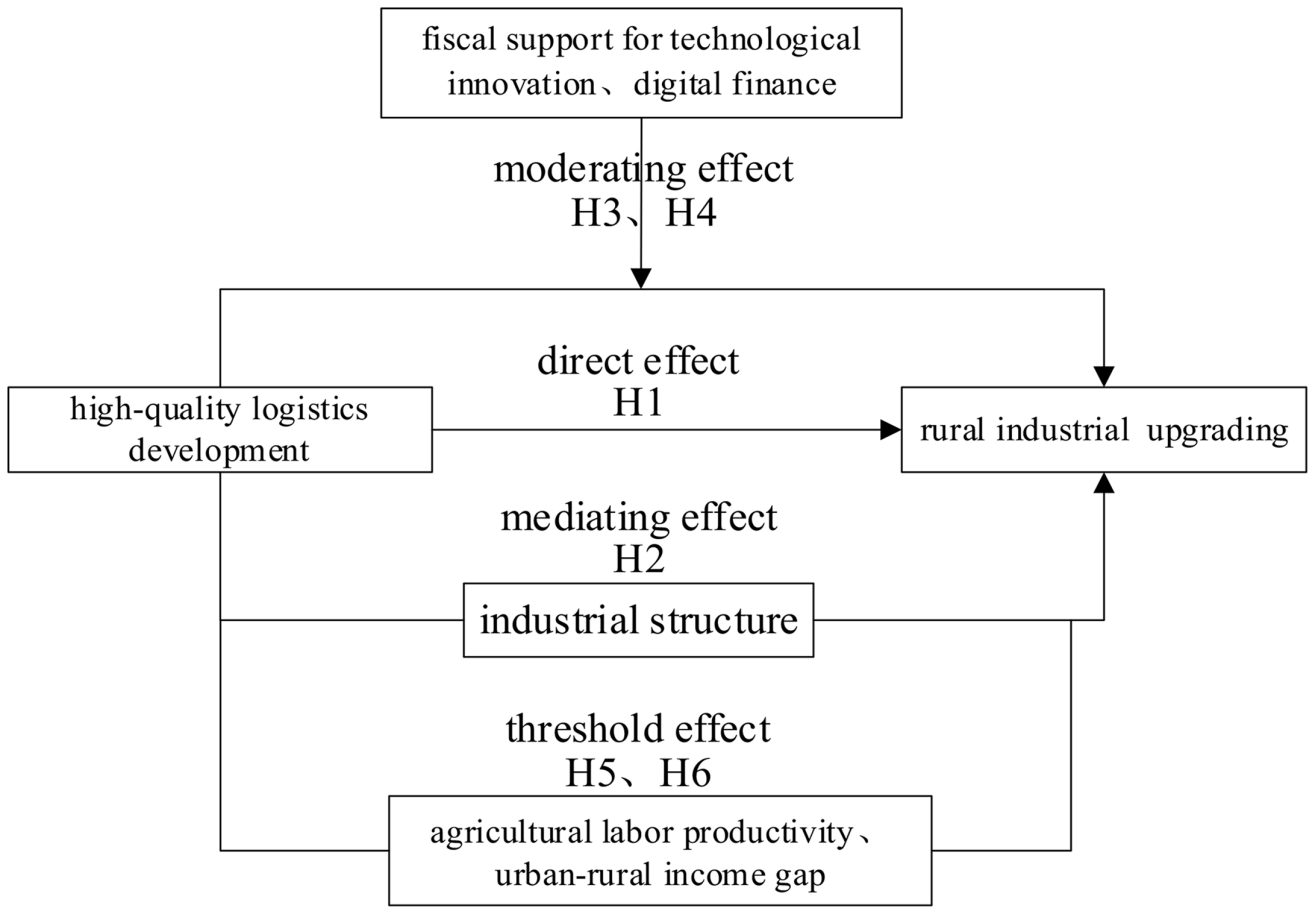
Theoretical Framework of the Mechanism Linking High-quality Logistics Development on Rural Industrial Upgrading.

The theoretical analysis framework of this paper is shown in Fig 1:

## 3 Methodology

### 3.1 Variable description and data sources

#### 3.1.1 Dependent variable and core independent variable.

The dependent variable in this study is rural industrial upgrading (nis), measured by the ratio of the total output value of services in agriculture, forestry, animal husbandry, and fishery (AFF) to the total output value of AFF. It directly captures the core of rural industrial transformation—the shift from low-value-added primary AFF production to high-value-added supporting services. These services inherently enhance value (e.g., through processing) and expand capabilities (e.g., via logistics), aligning with the objective of rural industrial upgrading. Importantly, it focuses on AFF-specific services rather than unrelated rural services such as retail, ensuring an accurate measure of upgrading within the rural agricultural ecosystem.

The core independent variable is high-quality logistics development (lh), measured using the input-output approach following Yan et al. [20]. Relevant indicators are selected from two dimensions: logistics industry inputs and outputs, and computed using the Super-SBM model, with detailed calculation procedures provided in S1 File. The input indicators include: (1) fixed asset investment in the logistics industry (100 million yuan), (2) number of employees (10,000 people), and (3) energy consumption (10,000 tons of standard coal). Due to the limited availability of logistics-specific data, information from the transportation, storage, and postal services industry is used as a proxy. Output indicators comprise the added value of the logistics industry (100 million yuan) and carbon emissions (Mt CO₂).

#### 3.1.2 Mediating variable.

The mediating variable is industrial structure level (is), represented by the proportion of the tertiary sector in GDP. high-quality logistics development promotes the optimization and upgrading of industrial structures, increasing the proportion of high-tech, high-value-added industries in rural areas while reducing the proportion of low-tech, low-value-added industries.

#### 3.1.3 Moderating variables.

The first moderating variable is regional fiscal support for technological innovation (rs), measured by the proportion of local government expenditure on science and technology in the general public budget. This indicator reflects the extent of government support for technological innovation and research activities. Higher fiscal support may enhance the positive effect of high-quality logistics development on rural industrial upgrading.

The second moderating variable is digital finance level (df), measured by the Digital Inclusive Finance Index compiled by the Institute of Digital Finance Peking University. This index comprehensively comprehensively captures the availability, usage, and impact of digital financial services, reflecting the role of digital finance in promoting economic development. A higher level of digital finance can accelerate the positive impact of high-quality logistics development on rural industrial upgrading.

#### 3.1.4. Threshold variables.

(1) Agricultural labor productivity (apl), defined as the agricultural output or agricultural value added per unit of labor over a given period. It is a key indicator of agricultural production efficiency, often associated with advances in agricultural technology and productivity.
(2) Urban-rural income gap (uri), measured as the ratio of urban residents’ per capita disposable income to rural residents’ per capita disposable income. This reflects the level of income disparity between urban and rural areas.

#### 3.1.5 Control variables.

(1) Economic development level (gdp), measured by real GDP, an essential indicator of a country or region’s total economic output, reflecting overall economic strength and development. Higher GDP typically implies better infrastructure, more active market demand, and stronger innovation capacity, which favor logistics development and indirectly support rural industrial upgrading. Additionally, economic growth may affect rural industrial upgrading through changes in residents’ consumption capacity, structure, and habits; for example, increased GDP may drive demand for high-value agricultural products, fostering deep processing and branding in rural industries.
(2) Rural residents’ per capita disposable income (irr), reflects rural living standards, economic strength, and purchasing power. Higher income may encourage investment in education, culture, and other non-material consumption, improving consumption patterns and indirectly supporting rural industrial upgrading. Increased income also boosts demand for high-quality agricultural products, promoting advanced rural industrial upgrading.
(3) Rural residents’ education, culture, and entertainment expenditure (ece), captures spending on intellectual and cultural activities, reflecting growing demand for cultural enrichment. This shift can drive rural industries toward diversified cultural products and services. Higher investment in education and culture enhances skills and innovation capacity, indirectly facilitating the transition of rural industries to technology-intensive and knowledge-intensive sectors.

#### 3.1.6 Data sources and descriptive statistics.

Due to data unavailability for Tibet, Hong Kong, Macau, and Taiwan, this study uses panel data from 30 Chinese provinces from 2011 to 2022. Data are primarily obtained from the China Statistical Yearbook, China Science and Technology Statistical Yearbook, and China High Technology Industry Statistical Yearbook. Missing data were supplemented using interpolation methods where necessary. The descriptive statistics of key variables are shown in Table 1.

**Table 1:** Descriptive Statistics.

Variables	Stats	N	Mean	SD	Min	Max	Units
Dependent variable	nis	360	0.043 2	0.020 3	0.013 1	0.131 4	
Independent variable	lh	360	0.370 0	0.272 4	0.103 8	1.398 6	
Mediating variable	is	360	0.496 7	0.089 5	0.326 6	0.838 6	
Moderating variables	rs	360	0.021 7	0.015 2	0.003 9	0.067 6	
	df	360	243.927 6	107.639 9	18.330 0	460.690 9	
Threshold variables	apl	360	3.349 4	1.756 2	0.384 1	11.018 4	10000yuan/person
	uri	360	258.500 0	42.797 4	183.000 0	398.000 0	
Control variables	gdp	360	2.400 5	1.985 2	0.128 7	10.240 7	Trillion yuan
	irr	360	1.395 8	0.632 4	0.427 8	3.972 9	10000yuan/person
	ece	360	10.578 9	3.393 8	4.457 0	23.423 8	100yuan/person

### 3.2 Model design

#### 3.2.1 Baseline model and mediating effect model.

To examine the direct effect of high-quality logistics development rural industrial upgrading, as well as the mediating role of industrial structure, the following recursive mediating model is constructed:


$${\text{nis}}_{i,t}=\alpha_{\text{0}}+\alpha_{\text{1}}{\text{lh}}_{i,t}+\alpha_{\text{2}}X_{i,t}+\mu_{i}+\gamma_{t}+\epsilon_{i,t}$$ (1)

$${\text{S}}_{i,t}=\alpha_{\text{0}}+\alpha_{\text{1}}{\text{lh}}_{i,t}+\alpha_{\text{2}}X_{i,t}+\mu_{i}+\gamma_{t}+\theta_{i,t}$$ (2)

$${\text{nis}}_{i,t}=\alpha_{\text{0}}+\alpha_{\text{1}}{\text{lh}}_{i,t}+\alpha_{\text{2}}{\text{S}}_{i,t}+\alpha_{\text{3}}X_{i,t}+\mu_{i}+\gamma_{t}+\sigma_{i,t}$$ (3)

Where the dependent variable nis represents the level of rural industrial upgrading, the core independent variable lh represents high-quality logistics development, and the mediating variable is the regional industrial structure level. X represents control variables. The subscripts *i* and *t* indicate region and year, respectively, while $\mu_{i}$ and $\gamma_{t}$ denote individual and time fixed effects, $\varepsilon_{i,t}$, $\theta_{i,t}$, and $\sigma_{i,t}$ represents the random disturbance term. Model (1) examines the direct effect of high-quality logistics development on rural industrial upgrading, while Models (2) and (3) test the mediating effect of regional industrial structure. To mitigate potential endogeneity concerns arising from the possible bidirectional relationship between logistics development (lh) and rural industrial upgrading (nis), this study employs a two-way fixed effects model that controls for both province-specific and time-specific unobserved heterogeneity. Although the model does not claim strict causal identification, it effectively reduces estimation bias caused by omitted time-invariant factors. The primary objective is to explore the interaction patterns and mechanisms between logistics development and rural industrial upgrading, rather than to establish causal direction. Future research may employ instrumental variable or dynamic panel approaches to further enhance causal inference.

Given that diagnostic tests strongly reject the null hypotheses of homoskedasticity (χ²(30) = 5479.10, p < 0.001) and no serial correlation (β = 0.844, t = 26.08, p < 0.001), we employ Driscoll-Kraay standard errors in all specifications to ensure robust inference.

#### 3.2.2 Moderation effect model.

To test whether regional fiscal support for technological innovation and digital finance level moderate the effect of high-quality logistics development on rural industrial upgrading, the following models are constructed:


$${\text{nis}}_{i,t}=\theta_{\text{0}}+\theta_{\text{1}}{\text{lh}}_{i,t}+\theta_{\text{2}}{\text{rs}}_{i,t}+\theta_{\text{3}}{\text{lh}}_{i,t}\times {\text{rs}}_{i,t}+\theta_{\text{4}}X_{i,t}+\mu_{i}+\gamma_{t}+\epsilon_{i,t}$$ (4)

$${\text{nis}}_{i,t}=\theta_{\text{0}}+\theta_{\text{1}}{\text{lh}}_{i,t}+\theta_{\text{2}}{\text{df}}_{i,t}+\theta_{\text{3}}{\text{lh}}_{i,t}\times {\text{df}}_{i,t}+\theta_{\text{4}}X_{i,t}+\mu_{i}+\gamma_{t}+\epsilon_{i,t}$$ (5)

Where, ${\text{rs}}_{i,t}\text{and}{\text{df}}_{i,t}$ represent the moderating variables, namely regional fiscal support for technological innovation and digital finance level, respectively, while other variables remain consistent with the previous models.

#### 3.2.3 Threshold effect model.

To further analyze the relationship between high-quality logistics development and rural industrial upgrading, it employs Hansen’s threshold regression model, using agricultural labor productivity (apl) and urban-rural income gap (uri) as threshold variables. If a single threshold exists, the following model is used:


$${\text{nis}}_{i,t}=\alpha_{\text{0}}+\alpha_{\text{1}}{\text{lh}}_{i,t}I({\text{apl}}_{i,t}\leq q)+\alpha_{\text{2}}{\text{lh}}_{i,t}I({\text{apl}}_{i,t}>q)+\alpha_{\text{3}}X_{i,t}+\mu_{i}+\gamma_{t}+\epsilon_{i,t}$$ (6)

Where, apl serves as the threshold variable, *I* (·) is an indicator function, and *q* represents the threshold value. When ${\text{apl}}_{\text{i},\text{t}}\leq \text{q}$, the effect coefficient of high-quality logistics development on rural industrial upgrading is $\alpha_{\text{1}}$; when ${\text{apl}}_{\text{i},\text{t}}>\text{q}$ the effect coefficient is $\alpha_{\text{2}}$; If $\alpha_{\text{1}}\neq \alpha_{\text{2}}$, a threshold effect exists; otherwise, it does not. If two thresholds exist, the model extends to the following double-threshold model:


$${\text{nis}}_{i,t}=\alpha_{\text{0}}+\alpha_{\text{1}}{\text{lh}}_{i,t}I({\text{apl}}_{i,t}\leq q)+\alpha_{\text{2}}\text{t}{\text{es}}_{i,t}I(q_{\text{1}}<{\text{apl}}_{i,t}\leq q_{\text{2}})+\alpha_{\text{3}}{\text{lh}}_{i,t}I({\text{apl}}_{i,t}>q_{\text{2}})+\alpha_{\text{4}}X_{i,t}+\mu_{i}+\gamma_{t}+\epsilon_{i,t}$$ (7)

where q_1_ and q_2_ are two threshold values, with q_1_ < q_2_. These two thresholds divide the sample into three regions. If three or more threshold values exist, this method can still be applied to construct multiple threshold models. Similarly, when the urban-rural income ratio (uri) serves as the threshold variable, the corresponding threshold model is:


$${\text{nis}}_{i,t}=\alpha_{\text{0}}+\alpha_{\text{1}}{\text{lh}}_{i,t}I({\text{uri}}_{i,t}\leq q)+\alpha_{\text{2}}\text{t}{\text{es}}_{i,t}I({\text{uri}}_{i,t}>q)+\alpha_{\text{3}}X_{i,t}+\mu_{i}+\gamma_{t}+\epsilon_{i,t}$$ (8)

## 4 Results

### 4.1 Baseline regression

Using STATA 17.0 software, we conducted an F-test and Hausman test on the baseline model, determining that a two-way fixed effects model is suitable for investigating the mechanism through which high-quality logistics development affects rural industrial upgrading. Table 2 presents the baseline regression results. Column (1) shows that, without considering control variables, high-quality logistics development significantly enhances rural industrial upgrading at the 1% statistical level. Column (2) adds a set of control variables, including economic development level, rural residents’ per capita disposable income, and rural residents’ expenditure on education, culture, and entertainment. Even after accounting for these controls, high-quality logistics development continues to exhibit a significant positive effect on rural industrial upgrading. This finding confirms that high-quality logistics development plays a pivotal role in promoting rural industrial upgrading, thereby validating Hypothesis 1.

**Table 2:** Baseline Regression Results.

Variables	(1)	(2)
	nis	nis
lh	0.016 6*** (0.006 2)	0.016 6*** (0.004 3)
gdp		0.008 2*** (0.000 4)
irr		0.020 3*** (0.003 0)
ece		0.001 3*** (8.50e-05)
Constant	0.031 2*** (0.002 0)	−0.006 7 (0.001 9)
id	Yes	Yes
year	Yes	Yes
Number of id	360	360
R-squared	0.282 3	0.484 7

Note: Robust standard errors (Driscoll-Kraay) are reported in parentheses.

* ** *** represents the significance levels of 10%,5% and 1% respectively.

### 4.2 Mediation effect test

As highlighted in the theoretical analysis, regional industrial structure may act as a critical mediating variable in the relationship between high-quality logistics development and rural industrial upgrading. The results of the mediation effect test are presented in Table 3. Columns (2) and (3) of Table 3 report the regression results with industrial structure as the mediating variable. Column (3) shows that industrial structure is positively significant at the 1% statistical level, indicating that high-quality logistics development contributes to the enhancement of regional industrial structure. Furthermore, after incorporating the mediating variable, the coefficient of high-quality logistics development decreases compared to the baseline regression, while industrial structure remains positively significant. This finding supports that industrial structure plays a mediating role in the relationship between high-quality logistics development and rural industrial upgrading. In other words, high-quality logistics development can promote rural industrial upgrading by enhancing regional industrial structure. Thus, Hypothesis 2 is validated.

**Table 3:** Mediation Effect Results.

Variables	(1)	(2)	(3)
	nis	is	nis
lh	0.016 6*** (0.004 3)	0.034 6*** (0.012 3)	0.013 9*** (0.003 8)
is			0.078 7*** (0.011 6)
gdp	0.008 2*** (0.000 4)	0.009 5*** (0.002 7)	0.007 4*** (0.000 3)
irr	0.020 3*** (0.003 0)	−0.006 2 (0.005 7)	0.020 8*** (0.003 4)
ece	0.001 3*** (8.50e-05)	−0.000 3 (0.000 2)	0.001 3*** (9.22e-05)
Constant	−0.006 7 (0.001 9)	0.412 0*** (0.007 8)	−0.039 2*** (0.005 5)
id	Yes	Yes	Yes
year	Yes	Yes	Yes
Number of id	360	360	360
R-squared	0.484 7	0.811 6	0.509 2

Note: Robust standard errors (Driscoll-Kraay) are reported in parentheses.

* ** *** represents the significance levels of 10%,5% and 1% respectively.

### 4.3 Moderating effect test

As discussed in the previous theoretical analysis, local fiscal support for technological innovation and regional digital finance levels may serve as two important moderating variables in the relationship between high-quality logistics development and rural industrial upgrading. To investigate this, we introduced these variables into the baseline model and analyzed the interaction terms between high-quality logistics development and local fiscal technological innovation support, as well as regional digital finance levels, to further explore the effect of logistics development on rural industrial upgrading. To eliminate the effect of multicollinearity, the interaction terms were mean-centered. The results of the moderating effect test are presented in Table 4. Column (1) shows the moderating effect of local fiscal technological innovation support. The results indicate that the interaction term between high-quality logistics development and local fiscal technological innovation support is significant at the 1% statistical level, and the signs of the core independent variable and the interaction term are the same. This suggests that local fiscal technological innovation support enhances the positive effect of high-quality logistics development on rural industrial upgrading, validating Hypothesis 3. Column (2) shows the moderating effect of digital finance level. The results indicate that the interaction term between high-quality logistics development and digital finance level is significant at the 1% statistical level, and the signs of the core independent variable and the interaction term are the same. This suggests that digital finance enhances the positive effect of high-quality logistics development on rural industrial upgrading, validating Hypothesis 4.

**Table 4:** Moderating Effect Test Results.

Variables	(1) rs	(2) df
	nis	nis
lh	0.018 5*** (0.004 7)	0.012 5*** (0.003 5)
rs	0.216 0** (0.120 0)	
lh_rs	0.601 0*** (0.099 8)	
df		0.000 3*** (2.28e-05)
lh_df		6.80e-05*** (7.01e-06)
Constant	−0.003 8** (0.001 7)	−0.000 2 (0.005 01 5)
id	Yes	Yes
year	Yes	Yes
Number of id	360	360
R-squared	0.514 8	0.540 7

Note: Robust standard errors (Driscoll-Kraay) are reported in parentheses.

* ** *** represents the significance levels of 10%,5% and 1% respectively.

### 4.4 Threshold effect test

In this study, we employed the bootstrap method with 1000 resampling iterations to examine the threshold effect of agricultural labor productivity. The results are shown in Table 5. The p-values for the single and dual threshold models with agricultural productivity as the threshold variable are 0.0070 and 0.6170, respectively. This indicates that the single threshold for agricultural productivity passes the significance test at the 1% statistical level, while the dual threshold does not pass the significance test. The single threshold value is 1.1516, with a 95% confidence interval of [1.1178, 1.1564].

**Table 5:** Threshold Effect Test Results — Agricultural Labor Productivity.

Threshold Variable	Threshold	Fstat	Prob	Crit10	Crit5	Crit1	Confidence Interval	
Agricultural Labor Productivity (apl)	Single	74.060 0	0.007 0	35.164 7	42.768 8	66.007 5	1.117 8	1.156 4
	Double	10.880 0	0.617 0	35.352 2	51.270 1	79.197 2	–	–

The estimation results for the single panel threshold are shown in Table 6. The regression results indicate that when agricultural labor productivity is low (apl ≤ 1.1516), the coefficient of the effect of high-quality logistics development on rural industrial upgrading is 0.1397. When agricultural labor productivity is higher (apl > 1.1516), the coefficient of the effect of high-quality logistics development on rural industrial upgrading is 0.0153. This disparity in coefficients reflects a diminishing returns pattern: regions with low agricultural labor productivity have greater potential for efficiency improvements in agricultural production and rural industrial operations, so high-quality logistics (e.g., optimizing agricultural product circulation, reducing factor mismatches) yields more substantial marginal gains for industrial upgrading. Conversely, regions with high agricultural labor productivity have already attained relatively efficient resource allocation, and thus the marginal contribution of logistics development to industrial upgrading is weakened. These findings suggest that high-quality logistics development exerts a relatively large effect on rural industrial upgrading when agricultural labor productivity is low, but a comparatively smaller effect when agricultural labor productivity is high. In other words, agricultural labor productivity exhibits a threshold effect in the relationship between high-quality logistics development and rural industrial structure upgrading, thereby validating Hypothesis 5.

**Table 6:** Threshold Regression Results — Agricultural Labor Productivity.

nis	Coefficient	Robust std. err.
gdp	0.007 3***	0.002 5
irr	−0.001 0	0.004 1
ece	0.000 5*	0.000 3
apl ≤ 1.1516	0.139 7**	0.054 3
1.1516 < apl	0.015 3*	0.008 5
_cons	0.015 7***	0.003 5
R-squared	0.481 3	
id	Yes	Yes
year	Yes	Yes

* ** *** represents the significance levels of 10%,5% and 1% respectively.

Similarly, the test for the case where the urban-rural income gap is used as the threshold variable is shown in Table 7. The p-values for the single and dual threshold models for the threshold variable are 0.0400 and 0.3890, respectively. This indicates that the single threshold for the urban-rural income gap passes the significance test at the 10% statistical level, while the dual threshold does not pass the significance test. The single threshold value is 363, with a 95% confidence interval of [360.0000, 371.0000].

**Table 7:** Threshold Effect Test Results — Urban-Rural Income Gap.

Threshold variable	Threshold	Fstat	Prob	Crit10	Crit5	Crit1	Confidence Interval	
Urban-Rural Income Gap (uri)	Single	44.890 0	0.040 0	32.057 6	41.597 5	63.878 7	360.000 0	371.000 0
	Double	17.280 0	0.389 0	49.167 0	70.725 0	121.753 9	–	–

The estimation results for the single panel threshold are shown in Table 8. The regression results indicate that when the urban-rural income gap is small (uri ≤ 363), that is, when the income disparity between urban and rural areas is low, the coefficient of the effect of high-quality logistics development on rural industrial upgrading is 0.0154. When the urban-rural income gap is large (363 < uri), the coefficient rises to 0.1377. This pattern reflects the “greater marginal gains in less developed areas” phenomenon: regions with a large urban-rural income gap often experience pronounced market segmentation and resource mismatches, and high-quality logistics can serve as a “bridge” connecting rural supply with urban demand, overcoming circulation barriers and more effectively unlocking the potential for rural industrial upgrading. Consequently, the promoting effect of high-quality logistics development is stronger in these regions than in regions with a small urban-rural income gap, where urban-rural integration is more advanced. This suggests that when the urban-rural income gap is small, the effect of high-quality logistics development on rural industrial upgrading is relatively limited, whereas when the gap is large, the effect is relatively large. In other words, the urban-rural income gap exhibits a threshold effect in the relationship between high-quality logistics development and rural industrial upgrading, thus verifying Hypothesis 6.

**Table 8:** Threshold Regression Results — Urban-Rural Income Gap.

nis	Coef.	Robust Std. Err.
gdp	0.007 3***	0.002 5
irr	−0.001 1	0.004 2
ece	0.000 5*	0.000 3
uri ≤ 363	0.015 4*	0.008 6
363 < uri	0.137 7**	0.052 3
_cons	0.015 4***	0.003 5
R-squared	0.479 2	
id	Yes	Yes
year	Yes	Yes

* ** *** represents the significance levels of 10%,5% and 1% respectively.

### 4.5 Robustness test

Although the bidirectional fixed effects model with Driscoll-Kraay standard errors used in the baseline regression mitigates endogeneity concerns and addresses heteroskedasticity and serial correlation, potential issues arising from omitted variables or reverse causality may persist. Therefore, it conducts robustness test using the following approaches (Table 9): First, an alternative indicator, ptes (Pure Technical Efficiency Score under Variable Returns to Scale), is employed to replace the comprehensive logistics efficiency measure as the independent variable (column (1)). Specifically, ptes captures the pure technical efficiency of the logistics industry, excluding scale effects, and reflects whether the industry optimally utilizes existing technologies to convert inputs into outputs, making it suitable for evaluating “management and resource allocation efficiency” independently of scale. Second, we include the lagged independent variable to further address reverse causality concerns (column (2)). Third, we implement province-clustered standard errors as an alternative inference method to verify the robustness of our statistical conclusions (column (3)). Fourth, we re-estimate our baseline model using Driscoll-Kraay standard errors to confirm the consistency of our main findings. Finally, we conduct a 1% winsorization treatment on all variables to mitigate the potential influence of outliers. The results across all specifications remain consistent with our baseline findings, confirming the reliability of our empirical conclusions.

**Table 9:** Robustness Test Results.

Variables	(1)	(2)	(3)
	nis	nis	nis
lh			0.016 6** (0.007 5)
ptes	0.016 3*** (0.002 4)		
L.lh		0.012 7*** (0.003 9)	
gdp	0.007 6*** (0.000 4)	0.008 5*** (0.000 4)	0.008 2*** (0.002 6)
irr	0.018 2*** (0.002 7)	0.021 8*** (0.003 5)	0.020 3 (0.012 8)
ece	0.000 9** (7.69e-05)	0.001 3*** (9.57e-05)	0.0013* (0.000 7)
Constant	−0.004 9** (0.002 1)	−0.046 0*** (0.006 1)	−0.024 3 (0.028 6)
id	Yes	Yes	Yes
year	Yes	Yes	Yes
Observations	360	330	360
Number of id	30	30	30
R-squared	0.526 2	0.459 9	–
Specification	Alternative measure (ptes)	Lagged independent variable	Province-clustered SE

Note:Robust standard errors (Driscoll-Kraay) are reported in parentheses.R-squared is not reported for the province-clustered specification in Column (3) due to computational differences in estimation methods.

* ** *** represents the significance levels of 10%,5% and 1% respectively.

## 5 Discussion and conclusion

### 5.1 Discussion

This study constructs an empirical model and a dual-moderation effect framework to investigate the collaborative relationship between high-quality logistics development and rural industrial upgrading. First, high-quality logistics development not only directly facilitates the optimization and upgrading of the rural industrial structure(H1), but also indirectly promotes rural industrial upgrading by enhancing regional industrial structure(H2). This aligns with previous studies emphasizing the positive role of logistics development in rural economic transformation. Moreover, the mediating role of industrial structure is consistent with the perspective of Yan et al. (2024) [4], while this study further clarifies the specific mechanism at work in rural contexts. Second, improvements in local fiscal support for technological innovation and digital finance levels provide strong incentives for deeper integration between logistics and rural industries, thereby amplifying the positive effect of high-quality logistics development on rural industrial upgrading. Although prior literature has acknowledged the moderating role of policy and financial factors [21], this study explicitly quantifies the moderating effects of local fiscal support for technological innovation (H3) and digital finance (H4). Finally, while most existing research on rural logistics have focused on linear relationships, this study highlights nonlinear dynamics through the threshold effects of agricultural labor productivity (H5) and the urban-rural income gap (H6). As threshold variables, agricultural labor productivity and the urban-rural income gap affect the magnitude of the effect of high-quality logistics development on rural industrial upgrading across different levels, indicating that regional heterogeneity and development stage characteristics should be fully considered when promoting the integration of logistics and rural industries.

### 5.2 Conclusion

This study extends the theoretical framework of rural industrial upgrading by integrating multiple effect paths, including direct, mediating, moderating, and threshold effects, and clarifies how factors like agricultural labor productivity and the urban-rural income gap constrain the effect of logistics. The mediating role of industrial structure highlights the unique urban-rural integration challenges in developing economies, offering valuable insights for comparable regions (such as Southeast Asia and sub-Saharan Africa). The findings also provide practical implications for policy-making. For government, priority should be given to supporting fiscal subsidies for rural logistics research and development and enhancing digital financial infrastructure, such as subsidizing smart warehousing and promoting mobile payment systems in rural areas. The observed threshold effects underscore the need for “phased” policies: in regions with low agricultural productivity, emphasis should be placed on fundamental logistics infrastructure (such as road network construction); whereas in regions with a large urban-rural income gap, logistics development should focus on connecting urban and rural markets. For enterprises, investment strategies should align with local conditions. In low-productivity regions, bulk transportation (such as grain and raw materials) offers higher returns, while in regions with a large urban-rural income gap, e-commerce logistics (such as rural terminal distribution) can unlock latent market potential.

Based on these findings, a three-tier progressive policy system: First, at the infrastructure level, an “intelligent logistics Hub+” action plan should be implemented, constructing national-level cold chain logistics bases for agricultural products to expand digital logistics coverage in county areas. Second, at the factor allocation level, a combination tool of “technology innovation vouchers digital inclusive finance” should be developed, establishing a rural revitalization technology special fund and promote blockchain-based agricultural product supply chain finance products. Lastly, at the institutional innovation level, a “differentiated evaluation-dynamic compensation” mechanism should be established, with special transfer payments for logistics infrastructure construction in regions with low agricultural labor productivity and pilot logistics revenue reinvestment mechanisms in regions with larger urban-rural income gaps. Critically, a collaborative governance platform should be built linking agricultural and rural authorities with financial regulators, integrating digital finance infrastructure into the development and evaluation of new agricultural business entities, thereby creating a sustainable ecosystem of “technology penetration-capital circulation-industry value addition.”

Similar evidence is found in other emerging economies. For example, in India, Negi and Negi (2022) examined humanitarian logistics challenges in disaster relief and proposed a framework for effectively managing supply chain operations, demonstrating that well-organized logistics enhances coordination, efficiency, and resilience in rural and vulnerable areas [22]. These findings support our conclusion that logistics development plays a critical role in promoting rural industrial upgrading, although the dual moderating effects of technological innovation and digital finance observed in China remain largely underexplored in other contexts.

### 5.3 Limitations and future directions

This study has several limitations. First, data from rural areas in China may not be fully generalizable to countries with mature rural economies, such as those in the European Union or the United States. Second, the relatively coarse granularity of rural micro-data may underestimate heterogeneous effects across regions or subpopulations. Additionally, the possible omission of subtle dynamics (e.g., how small farmers specifically benefit from logistics) may affect the accuracy of policy recommendations.

Moreover, the use of provincial-level data may obscure intra-provincial heterogeneity, including variations in infrastructure density and accessibility to digital financial services. It should be explicitly acknowledged that due to the observational nature of the data and the lack of a valid instrumental variable, strict causality cannot be fully confirmed, and the results of this study should be interpreted as robust associations. Future research could address these limitations by employing more granular datasets or conducting regional case studies, thereby enabling a deeper analysis of the relationship between logistics development and rural industrial upgrading, providing more targeted guidance for global rural revitalization efforts.

## Supporting information

S1 File **S1 Fig.** First Threshold Regression Results — Agricultural Labor Productivity. **S2 Fig.** First Threshold Regression Results — Urban-Rural Income Gap. **S1 File.** Measurement Index System of High-Quality Development Level of Logistics Industry.
(RAR)
